# Medical aid to war victims in Syria in 2019: a report of organized healthcare support from a charity organization

**DOI:** 10.1186/s12913-022-08507-z

**Published:** 2022-09-10

**Authors:** Łukasz Przepiórka, Mariusz Boguszewski, Cezary Smuniewski, Sławomir Kujawski

**Affiliations:** 1 Department of Neurosurgery, Medical University of Warsaw, Warsaw, Poland; 2 Faculty of Theology, Cardinal Stefan Wyszynski University in Warsaw, Warsaw, Poland; 3 University of Warsaw, Warsaw, Poland; 4 Department of Exercise Physiology and Functional Anatomy, Ludwik Rydygier Collegium Medicum in Bydgoszcz Nicolaus Copernicus University in Torun, Bydgoszcz, Poland

**Keywords:** War victims, Report, Healthcare support, Syria

## Abstract

**Background:**

In 2011, a civil war started in Syria, which is on-going and has reached a death toll of over 400,000 people. Humanitarian organizations, including Aid to The Church in Need (ACN), have strived to provide help and medical support to the civilian victims.

**Methods:**

We performed a retrospective analysis of data gathered in ACN projects in Syria in 2019. The datasets included descriptions of diseases, treatments, costs, cities, and hospitals. For each patient, we assigned the following additional categories: type of help (treatment, diagnosis, or nonmedical), type of treatment (medical or surgical), medical specialty, gross anatomic region, and presence of trauma.

**Results:**

A total of 3835 patients benefited from ACN support in Syria in 2019. The majority of financial support went towards treatment (78.4%), while other support went towards nonmedical help (15.7%) or providing a diagnosis (5.9%). Among treatments, 66.6% were medical and 33.4% surgical. The most common medical specialty was internal medicine (48.4%), followed by public health (13.7%) and surgery (7.3%). Anatomic region was undefined in 68.3% of cases and, when defined, was most commonly the abdominal cavity and pelvis (13%). The vast majority of cases 95.1%) were not associated with trauma. Procedural costs were highest in the Valley of Christians region, and lowest in Tartous. Network graphs were used to visualize the three most common diagnoses and treatments for each medical specialty.

**Conclusions:**

The present report describes the treatment of war victims in Syria in 2019. The patients lacked the most basic medical or surgical healthcare. Charity organizations, like ACN, constitute a valuable source of information about the healthcare of war victims. Unfortunately, the methods of describing medical treatment provided to civilian victims remain underdeveloped. Future studies will require the cooperation of healthcare providers, humanists, and social workers. The present findings can help to optimize the provision of humanitarian help by charity organizations, by tailoring projects to the specific needs of Syrian war victims.

## Introduction

In 2011, “the Arab Spring” uprisings reached Syria, starting a civil war that has not yet ended.

The death toll is over 400,000 people, including around 20,000 children, and millions have been forced to leave their houses, leading to a refugee crisis [1]. Since the start of the conflict, multiple humanitarian organizations have closely observed the civilian suffering. For instance, Aid to The Church in Need (ACN) has played a major role in spreading news about the humanitarian disaster and genocide in Syria, while also providing help and medical support to the victims. ACN is a Pontifical Foundation of the Catholic Church, which supports all people, regardless of their faith. In the case of the Catholic faithful and other Christians where they are prosecuted, oppressed, or in pastoral need. Each year, ACN funds projects around the world, including stipends, construction and reconstruction projects, and the provision of medical aid [2].

In the present study, we aimed to describe and analyze data regarding the healthcare support provided to patients in Syria by ACN in 2019. In this context, we endeavored to illustrate the problems faced in providing organized help to the war victims, and to suggest solutions where possible. Finally, we discuss directions for future studies about the provision of healthcare support to war victims.

## Methods

### Materials

We performed a retrospective analysis of the data gathered in ACN projects in Syria in 2019. For each project, a local ACN council—comprising a medical doctor, social worker, and parish (or foundation) worker—decides whether the patient requires financial aid and, if so, the percentage of its coverage. The decision is based on the following inclusion criteria: being a war victim, poverty (determined by the local council), and preexisting need for medical treatment. There are no exclusion criteria. Importantly in such a religiously diverse region, the local council does not consider religion and denomination. Methods of aid can include partially or fully paying for the treatment, with the local council deciding the percentage of treatment coverage.

### Data collection

ACN provided the anonymized data including financial (cost per treatment) and medical (type of diagnosis and therapy) information. Raw data were prospectively collected during each ACN project carried out in Syria in 2019, at hospitals located in Aleppo, Tartous, Damascus, and Marmarita (Homms).

Damascus, capital of Syria, with an estimated population of 2 million people in 2008 is the most important city accommodating national administration. It was heavily damaged during war and became shelter for many refugees from neighboring warzones. Aleppo, the largest city of Syria, is located near the Turkey border. Between 2012 and 2016, a major confrontation, known as battle of Aleppo, took place, yet some lesser fights continued afterwards. Tartous is located near the Libyan border on west of Syria. It suffered minimal damage during war and offered a sanctuary for many internal refugees as well as people awaiting departure from Syria to Lebanon or, through Lebanon, to other locations. Marmarita is located approximately 60 km from Homs. It avoided much of the warfare and offered shelter for many refugees from Aleppo and other Syrian cities. Homs is located 162 km north from Damascus and has population of approximately 600 thousand people.

For each patient assistance (i.e. provision of financial support to a specific patient with a specific medical condition), there was a description including the disease, treatment, date, group, costs, city, and hospital. These reports were translated into English, categorized using the methods described below, and analyzed. Cost of treatments was initially provided in Polish zloty (PLN). To increase clarity of data to a wide spectrum of recipients, we have converted currency to United States Dollar (USD) based on the mean currency from 2019. Data analysis was influenced by the guidelines for reporting economic evaluations – Consolidated Health Economic Evaluation Reporting Standards (CHEERS) [3]. We did not adhere to these guidelines because economic evaluation was not the aim of the study. Additionally, economic evaluations, by the definition, analyze “alternative courses of action in terms of their costs and their consequences” [4]. Due to no follow–up in our study, a proper economic evaluation cannot be performed.

### Categorization (classification of data concerning the provision of medical aid)

To our knowledge, there is presently no standard classification method for data concerning the provision of medical aid to war victims. To overcome this lack, we designed the following system to present details regarding Syrian patients. We developed additional categories based on the descriptions of diseases and treatments for each patient assistance in the available database. Firstly, each patient assistance was categorized as a “treatment”, a “diagnosis”, or “nonmedical”. Any notes containing depiction of a medical or surgical treatment were assigned to the “treatment” category. Notes containing no description of medical intervention, but depictions of diagnostic procedures were assigned to the “diagnosis” category. The third category, “nonmedical”, included types of help that were not strictly associated with hospital admission, outpatient appointment, etc.—for instance, buying medical equipment or therapeutic sessions. All patients in the “treatment” category were assigned a subcategory of “medical” or “surgical”, based on the nature of the specific help provided. Subsequently, each patient assistance, based on the description of the diagnosis or treatment, was categorized as being within one medical specialty. When possible, a gross anatomic region was assigned, i.e. head and neck, upper limb, lower limb, thorax, abdominal cavity, or undefined/not applicable. Finally, each assistance was described as “traumatic” or “nontraumatic” according to whether there was a clear description of trauma. All categorizations were performed by a single author (ŁP), who is a medical doctor. Single author categorization asserted homogeneousness throughout the process.

### Data analysis

The economic evaluation and statistical analysis were conducted on data that included currency in USD and additional variables with categories regarding medical aid described above. The Mann-Whitney U test was used to compare costs in USD between patients with and without trauma.. Differences in cost of treatment are presented as median ± *interquartile range* (*IQR*). In addition, sums and means of a treatment cost for specific geographical regions are provided. Qualitative data (type and quantity of specific treatments, medical specialty, anatomic region, presence of trauma, prevalence in cities) were presented as count and percentage. Network graphs were created using Cytoscape software version 3.8.1 [5]. Network graphs were used to present the most common medical diagnoses and medical treatments per specialty assigned to patients. Three most common categories were shown in each graph to increase its clarity. Statistical investigation was performed using Statistica 13.0 (StatSoft, Inc.). All analyses were performed with level of α = 0.05.

## Results

**Fig. 1:**
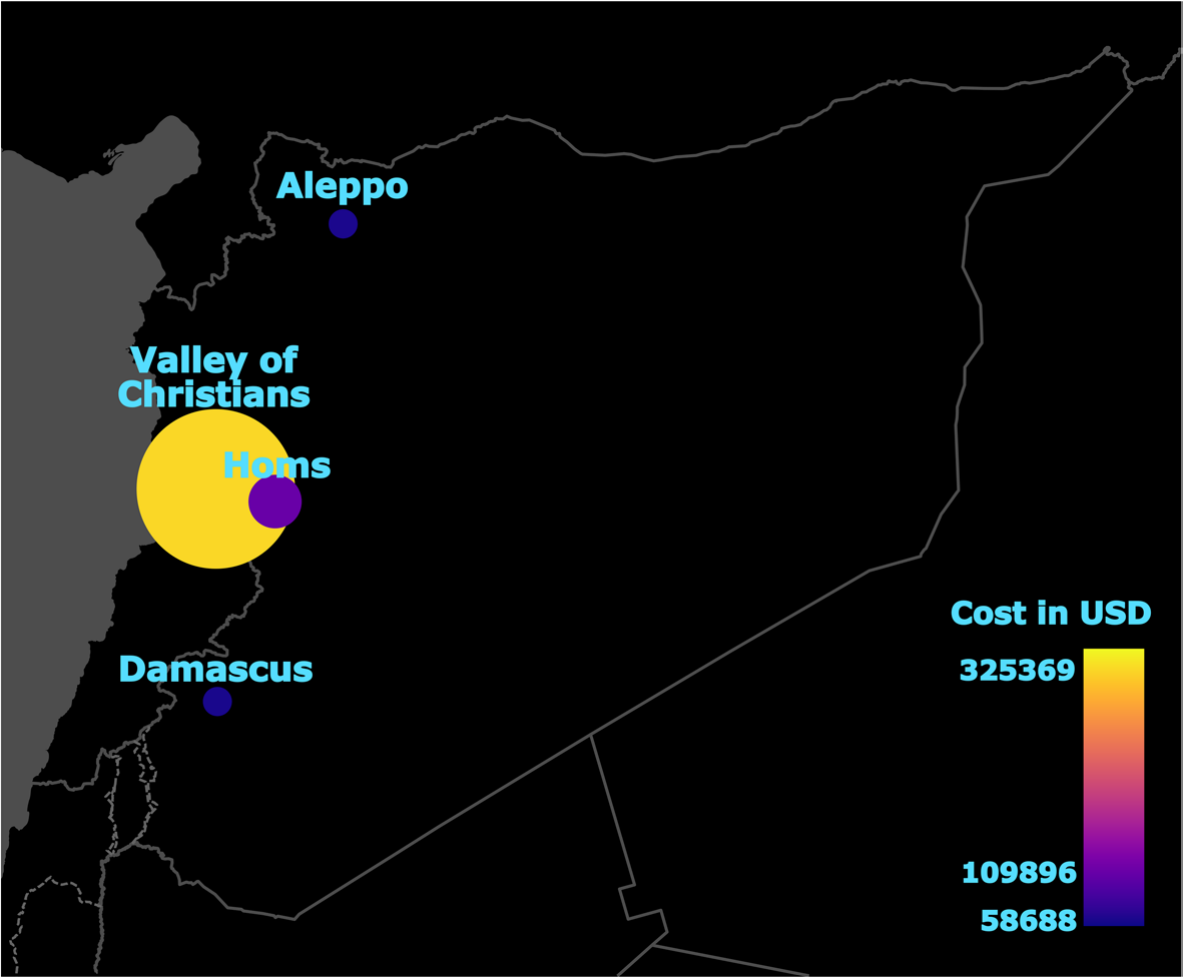
Bubble map of Syria presenting the costs of medical procedures according to region. The size of bubbles near the name of a location is proportional to the cost of medical procedures (in USD). Additionally, the cost in USD is color-coded in the plasma palette, with the lowest cost denoted by blue and the highest by yellow

A total of 3835 patients benefited from ACN support in Syria in 2019. A gross majority of the financial support provided was focused on treatment (78.4%), while the rest went towards nonmedical support (15.7%) or providing a diagnosis (5.9%). Among the provided treatments, two-thirds (66.6%) were medical and one-third (33.4%) surgical. In terms of medical specialty, patient assistances were most commonly categorized as internal medicine (48.4%), followed by public health (13.7%) and surgery (7.3%). Anatomic region was undefined in over two-thirds of cases (68.3%) and, when defined, was most commonly the abdominal cavity and pelvis (13%), followed by head and neck (8%) and thorax (7.5%). The vast majority of cases were not associated with a trauma (95.1% vs 4.9%). The cost of treatment was lower for patients without trauma than for patients with trauma (median ± IQR: 59.75 ± 130.6 USD vs 350.96 ± 374.6 USD, respectively; Z = −10.40, *p* < 0.0001). Details regarding the different categories are presented in Table 1 and Fig. 1.

**Table 1:** Overview of healthcare aid provided to patients in Syria in 2019 by Aid to the Church in Need

Category	Data
**Type of help**	
Treatment	3008 (78.4%)
medical	2003 (66.6%)
surgical	1005 (33.4%)
Diagnosis	224 (5.9%)
Nonmedical	603 (15.7%)
**Medical specialty**	
Cardiology	184 (4.8%)
Internal medicine	1859 (48.4%)
Neurology	139 (3.6%)
Neurosurgery	39 (1%)
Obstetrics and gynecology	236 (6.2%)
Oncology	97 (2.5%)
Ophthalmology	181 (4.7%)
Orthopedics	172 (4.5%)
Otorhinolaryngology	78 (2%)
Pediatrics	14 (0.4%)
Psychiatry	22 (0.6%)
Public health	526 (13.7%)
Radiology	8 (0.2%)
Surgery	280 (7.3%)
**Anatomic region**	
Head and neck	305 (8%)
Thorax	289 (7.5%)
Abdominal cavity and pelvis	500 (13%)
Upper limb	31 (0.8)
Lower limb	92 (2.4%)
Undefined / not applicable	2618 (68.3%)
**Trauma**	
Yes	186 (4.9%)
No	3649 (95.1%)
**City**	
Aleppo	159 (4.1%)
Damascus	121 (3.2%)
Homs	707 (18.4%)
Tartous	4 (0.1%)
Valley of Christians	2844 (74.2%)
**Costs**	
Total	236,254.75
Mean	583.12
Median	240.86

Procedure costs were highest in the Valley of Christians region (325369USD for 2844 procedures; ~114,41USD per procedure), and lowest in Tartous (18,460 USD for 4 procedures in overall. However, mean cost per procedure was the highest in Tartous from all examined regions (~4614,88 per procedure). The next region with the lowest sum of costs was Damascus (58,688 USD for 120 procedures in overall) with the second of the highest mean cost of procedure (~489,06 USD per procedure). Sum of costs in Homs was 109,896 USD, while in Aleppo it was 59,865 USD. The overall number of procedures was 3741 with total cost of 572,278 USD (Fig. 1). Figure 1 does not show Tartous, due to the lower number of health care assistances provided there (Table 1).

To analyze the full spectrum of diseases and treatments is beyond the limits of this paper. Therefore, to represent the database, we provided three most common diagnoses and treatments for each medical specialty (Table 2). Careful analysis reveals the diagnoses and treatments that are most conventional for each specialty, and not particularly difficult. None of the diseases in the database could be described as complex or requiring an academic center.

**Table 2:** Three most common diagnoses and treatments for each medical specialty

Medical specialty	Three most common diagnoses, number (percent in specialty)	Three most common treatment descriptions, number (percent in specialty)
Cardiology *n* = 184	Angina pectoris, 121 (65.8%) Myocardial infarction, 23 (12.5%) Cardiac problems, 10 (5.4%)	Surgery to install cardiac catheter and drug eluted cardiac stent, 67 (36.4%) Surgery to install cardiac catheter, 38 (20.7%) Cardiac catherization percutaneous coronary intervention, 28 (15.2%)
Internal medicine *n* = 1859	The patient is suffering from: hypertension, high cholesterol, 518, (27.9%) The patient is suffering from: hypertension, high cholesterol, diabetes, 157 (8.4%) The patient is suffering from: hypertension, 110 (5.9%)	Medications, 1683 (90.5%) Hospital admission, 88 (4.7%) Lab analysis, 27 (1.5%)
Neurology *n* = 139	The patient is suffering from: Epilepsy, 51 (36.7%) The patient is suffering from a stroke, 7 (5%) The patient is suffering from cerebral palsy, 5 (3.6%)	Medications, 83 (59.7%) MRI, 14 (10.1%%) Physical therapy, 11 (7.9%)
Neurosurgery *n* = 39	The patient is suffering from problems with spinal vertebrae, 20 (51.3%) Basocervical fracture, 3 (7.7%) Brain tumor, 3 (7.7%)	Surgery of resection the tumor, 5 (12.8%) Anchorage, 4 (10.3%) MRI, 3 (7.7%)
Obstetrics and gynecology *n* = 236	The patient nine months pregnant, 125 (53.0%) Breast Cancer Awareness, 24 (10.2%) Cesarean section, 20 (8.5%)	Cesarean section, 154 (65.3%) Hysterectomy surgery, 27 (11.4%) Knowledge ways to detect breast Cancer, 24 (10.2%)
Oncology *n* = 97	The patient is suffering from breast cancer, 21 (21.6%) The patient is suffering from benign prostatic hyperplasia, 15 (15.4%) The patient is suffering from breast tumor, 11 (11.34%)	Medications, 35 (36.1%) Surgery of resection the tumor, 9 (9.3%) Mastectomy, 9 (9.3%)
Ophthalmology *n* = 181	The patient is suffering from cataract, 99 (54.7%) The patient is suffering from myopia, 22 (12.2%) The patient is suffering from retinopathy, 7 (3.9%)	Surgery of phaco and intra ocular lens, 91 (50.3%) Medications, 42 (23.2%) Medical glasses, 25 (13.8%)
Orthopedics *n* = 172	The patient is suffering from a broken femoral bone, 23 (13.4%) The patient is suffering from a broken leg, 13 (7.6%) The patient is suffering from degenerative joint, 13 (7.6%)	Medications, 66 (38.4%) Open reduction internal fixation surgery, 22 (12.8%) Surgery for internal bone fixation with rods implants, 18 (10.5%)
Otorhinolaryngology *n* = 78	The patient is suffering from tonsillitis, 16 (20.5%) The patient is suffering from deviated nasal septum, 12 (15.4%) The patient is suffering from hearing deficiency, 9 (11.5%)	Tonsillectomy, 21 (26.9%) Nasal septal deviation surgery, 12 (15.4%) Medical earphone, 7 (9.0%)
Pediatrics *n* = 14	The patient is suffering from growth deficiency, 4 (28.6%) The newborn baby is suffering from hypoxia, 2 (14.3%) */all other were singular/*	Hospitalization and incubator, 8 (57.1%) Lab analysis, 2 (14.3%) Medications, 2 (14.3%)
Psychiatry *n* = 22	The patient is suffering from: depression, 8 (36.3%) The patient is suffering from: depression, insomnia, 3 (13.6%) The patient is suffering from: ADHD, 2 (9.1%)	Medications, 21 (95.4%) Electroconvulsive therapy, 1 (4.6%) */no other options/*
Public health *n* = 526	Children with a disability (as a result of war or a congenital disability) at home pose a major challenge for their parents, on the one hand they accept the condition of their children, and society accepts them, 136 (25.9%) We have some cases of violent behaviors, sometimes expressed by beating, the reason is what they have experienced during the war., 93 (17.7%) Spreading the message of peace between volunteers of the association by participating in painting and coloring on the walls, 50 (9.5%)	The program helps parents to accept their handicapped child at home and to be freed from the estimation of community and not feeling ashamed showing him/her, offering educational courses and awareness sessions, which help them deal with their child at home. 183 (34.8%) Individual and collective sessions were done to children through some meaningful activities, games, plays, music and free drawing which help children discharge their energy., 93 (17.7%) Volunteers implement the activity by drawing on the garden walls reflecting the association values. 50 (9.5%)
Radiology *n* = 8	Carcinoma, 3 (37.5%) PET scan, 2 (25%) */all other were singular/*	CT scan, 6 (75%) PET scan, 2 (25%) */no other options/*
Surgery *n* = 280	Inguinal hernia, 30 (10.7%) The patient is suffering from acute cholecystitis, 21 (7.5%) The patient is suffering from biliary inflammation, 17 (6.1%)	Inguinal hernia repair surgery, 25 (8.9%) Cholecystectomy, 25 (8.9%) Lithotripsy, 21 (7.5%)

Responses are presented as they appeared in the database. In certain cases, there were only two types of descriptions for the whole specialty. In some cases, only two types of descriptions were multiple occurrences, while all others were single instances. In those two scenarios, only two answers are provided

*ADHD* Attention-deficit hyperactivity disorder, *CT* Computed tomography, *MRI* Magnetic resonance imaging, *PET* Positron emission tomography

**Fig. 2:**
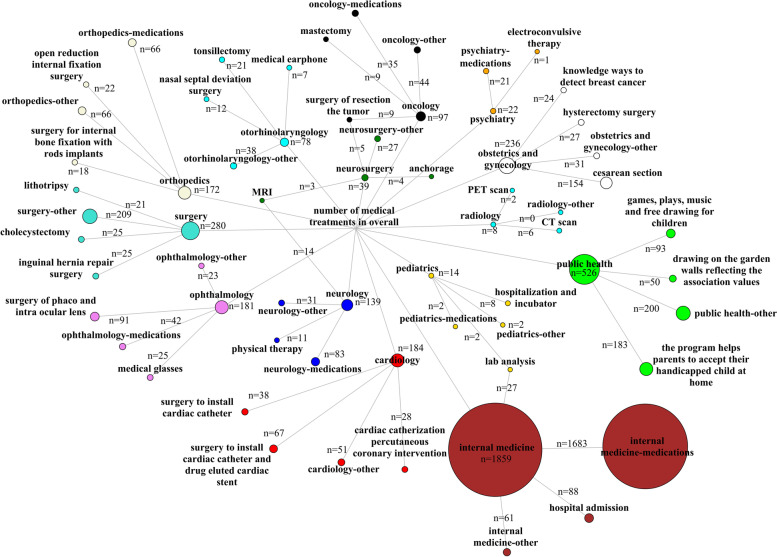
Network graph presenting the three most common diagnoses per specialty assigned to patients in Syria in 2019 by Aid to the Church in Need. The medical specialty categories are indicated by node color: cardiology (red), internal medicine (brown), neurology (blue), neurosurgery (green), obstetrics and gynecology (white), oncology (black), ophthalmology (violet), orthopedics (beige), otorhinolaryngology (cyan), pediatrics (gold), psychiatry (plum), public health (lime), and radiology (aqua). The node size (shows as dots) is proportional to the number of diagnoses. Edges (connections between nodes) are shown in grey. ADHD – attention-deficit hyperactivity disorder, CT – computed tomography, MRI – magnetic resonance imaging, PET – positron emission tomography

**Fig. 3:**
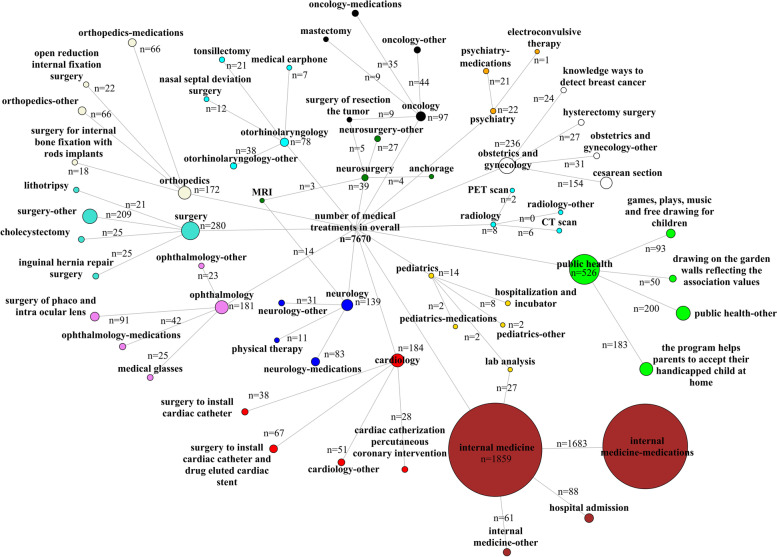
Network graph presenting three most common treatments per specialty provided to patients in Syria in 2019 by Aid to the Church in Need. The medical specialty categories are illustrated by node color: cardiology (red), internal medicine (brown), neurology (blue), neurosurgery (green), obstetrics and gynecology (white), oncology (black), ophthalmology (violet), orthopedics (beige), otorhinolaryngology (cyan), pediatrics (gold), psychiatry (plum), public health (lime), and radiology (aqua). The node size (shows as dots) is proportional to the number of treatments. Edges (connections between nodes) are shown in grey. ADHD – attention-deficit hyperactivity disorder, CT – computed tomography, MRI – magnetic resonance imaging, PET – positron emission tomography

The relatively low percentages among most common descriptions indicate a wide variety of responses. For a handful of specialties, only two diagnoses or treatments are described. This is for two reasons: a lack of more responses, or the finding that all other responses had the same frequency of appearance and a third most common could not be identified. The three most common diagnoses and treatments are presented as network graphs in Figs. 2 and 3, respectively.

Reviewing the descriptions from the database revealed several reappearing problems. Firstly, many of the descriptions lacked sufficient medical expertise and seemed to be recorded by a non-medical translator, which affected the language quality. Secondly, the diagnosis and treatment descriptions often contained the same information, e.g. a cesarean section in obstetrics and gynecology that appeared in both categories. Thirdly, some descriptions were specific, while others were general, e.g. “medications”. Finally, in numerous cases, a particular diagnosis or treatment was described in several different ways—for example as “surgery to repair hernia” and “hernia repair surgery” (Table 2). Such repetition impeded categorization, and made more detailed analysis impossible.

The collected data were derived from all major hospitals in Syria—namely, those located in Aleppo, Tartous, Damascus, and Marmarita (Homms). Importantly, other hospitals are not significant sources of providing specialized medical healthcare, and often do not employ medical doctors or nurses. Therefore, our database provides a reliable report of treatments provided to the poorest and neediest patients in Syria.

## Discussion

In this study, we analyzed data gathered by ACN during their medical aid projects supporting war victims in Syria in 2019. We introduced a simple categorization scheme to describe healthcare provided to war victims, which could be used by future researchers. We also identified the main advantages and disadvantages of the analysis of medical charts from charity organizations that provide healthcare support to war victims.

### Research on medical aid to Syrian war victims

There are limited data available regarding medical aid provided to Syrian war victims in the setting of ongoing armed conflict. Many reports describe medical aid provided to Syrian refugees in other countries where they have found refuge [6–18]. However, this is fundamentally different from providing healthcare within a country under civil war, and the differences extend far beyond the setting of the healthcare system [19]. Compared to places without conflict, warzones face many problems, e.g. deliberate attacks on healthcare facilities in Syria, preventing healthcare workers from receiving education [20–28], and the reported use of chemical weapons [29, 30]. As a result, Syrian healthcare system has been significantly damaged [31–34]. Noteworthy, in 2012 the World Health Organization (WHO) reported only half of hospitals in Syria functional [35]. Furthermore, the level of care provided by medical facilities in developing countries is far below that provided in high-income countries. Finally, a follow–up for war victims in the setting of ongoing war is usually unobtainable. For instance, Fatima et al. analyzed over 41 thousand neurotrauma cases from 95 war-affected hospitals in Syria from 2013–2015, however their medical data was limited to the hospitalization period [36].

### Novelty of studies of charity projects

Most articles describing treatment of war victims focus either on a specific medical specialty [37–41], or on a certain group of diseases [8, 42–48], commonly PTSD [14, 49]. Surgical trauma case series are a special group of interest in the literautre [50–53]. Some articles report a single-center experience [14, 54, 55] and some describe creating local clinics and facilities founded by outside sources [56]. Therefore, our manuscript introduces novelty to the literature and opens space for future research. Notably, there have been reports, and even systematic reviews, of the provision of cash-based assistance for people who live in the area of conflict [57, 58]. However, such assistance is essentially different from the provision of medical support as presented herein.

Studies of charity projects require the cooperation of people with various sets of talents, including healthcare providers, humanists, and social workers. No previous study has described the medical treatment of war victims in Syria in a comprehensive manner. Our presently utilized system of categorization is straightforward and could be reproduced and improved. However, a simpler and better solution would be to use already existing medical classification coding list for diseases—e.g. International Statistical Classification of Diseases and Related Health Problems (ICD) by the WHO—and procedure codes (e.g. ICD–10–CM). Proper reporting and understanding of the healthcare needs of war victims could enable charity organizations to undertake more tailored projects and optimize their use of resources [59].

### Ethical necessity of supporting war victims

A brief analysis of the database reveals that healthcare support provided to war victims in Syria covers the most common and fundamental medical and surgical treatments among all specialties (Table 2). The authors did not identify any procedures that could be described as complex cases. This clearly illustrates that war victims in Syria are lacking some crucial medical procedures. Consequently, complex surgical cases are treated outside Syria [52] or with a help of foreign physicians [60]. Furthermore, many of the diagnoses are considered life-threatening and requiring urgent treatment, e.g. myocardial infarction. Statistical analysis revealed that cost of treatment was significantly higher for patients with trauma than for those without trauma. Ideally, providing adequate healthcare to war victims should be an area of interest and action for high-income countries outside of an armed conflict.

In 2009 there were 1485 organizations (including charitable organizations) registered with the Ministry of Social Affairs and Labour in Syria [61]. United Nations High Commissioner for Refugees Operational Portal lists 28 partners, “including government ministries, international non-governmental organisations (NGOs), national NGOs and UN agencies” [62]. Some of organizations that took action in Syria are faith-based organisations (FBOs), for instance Caritas, Islamic Relief, and World Vision [63]. Some authors noted that FBOs category is rather a fluid one, as no all organizations clearly establish their roots in faith. Additionally, some organizations became more secular over time [64]. A significant proportions of FBOs cooperate with national NGOs or other members of local civil society in Syria [63]. Motives of different FBOs organizations might differ because of variety of motives of charity in specific religions. FBOs grounded in Islam are related to “the *zakat*” one of commandments for Muslim that asks to care for the poor [65]. ACN action, as an embodiment of charity in Christianity, is inspired by *Agape*, which is a cardinal element of Christian faith, primarily defined as God’s own love towards people. “*Agape* (…) is caused only by Jesus Christ, who died for us while we were still sinners. (…) *Agape* goes beyond justice. It reaches out to the unjust as well, and creates good, with no personal gain forthcoming” [66]. All people around the world require adequate healthcare, and armed conflicts should not impede that. Notably, war generates many more patients. In addition to issues that require immediate help, other problems arise that are visible only in long–term follow–up [67]—for instance, increased war-related family violence [68–70] and impaired parent–child interactions among Syrian refugees [71].

### Limitations

The presented data are based on a database, which is an English translation of original files in Syrian; therefore, some information could have been lost in translation or be inaccurate. Furthermore, the database itself contains numerous internal insufficiencies that compromise its value, mostly related to the quality of medical descriptions or the English language. The current research primarily applied descriptive analysis in addition to comparison between groups of treatment costs. Due to sparse number of variables in database (e.g., no demographic patient data) models that would include those variables as co-factors could not be applied. Additionally, presenting treatment outcomes and follow–up should be performed in future studies.

## Conclusions

In this report, we describe the projects undertaken by ACN in Syria, providing a reliable and comprehensive source of information about the treatment of war victims in Syria in 2019. Despite the imperfections of the collected data, we can conclude that the war victims in Syria required the most common and basic medical and surgical procedures. Additionally, the cost of treatment was higher for trauma patients than in patients without trauma.

Charity organizations, like ACN, and their projects provide a valuable source of information about the healthcare provided to war victims. Such data, if it were carefully collected with the aid of healthcare professionals, could provide valuable insight into the current condition of war victims and their needs. For this reason, more detailed data collection is needed. Notably, the methods of describing medical treatment of war victims are still underdeveloped. There remains a need for future studies of charity projects, which will require cooperation of people with various sets of talents, including healthcare providers, humanists, and social workers. The potential benefit of such studies will be to help optimize the humanitarian aid provided by charity organizations, through tailoring their projects to the specific needs of Syrian war victims.

## Data Availability

The data supporting the findings of this study are available from Aid to the Church in Need. Restrictions apply to the availability of these data, which were used under license for the current study, and are thus not publicly available. However, data are available from the corresponding author upon reasonable request and with permission of Aid to the Church in Need.

## Abbreviations

ACN: Aid to the Church in Need
FBO: Faith-based organisation,
NGO: Non-governmental organization
